# Is double-strut fibula ankle arthrodesis a reliable reconstruction for bone defect after distal tibia tumor resection?—a finite element study based on promising clinical outcomes

**DOI:** 10.1186/s13018-021-02362-0

**Published:** 2021-03-29

**Authors:** Zhiqing Zhao, Taiqiang Yan, Wei Guo, Rongli Yang, Xiaodong Tang

**Affiliations:** Musculoskeletal Tumor Center, Peking University People’s Hospital, No. 11 Xizhimen South Street, Xicheng District, Beijing, 100044 China

**Keywords:** Bone tumor, Distal tibia, Fibular graft, Finite element analysis

## Abstract

**Background:**

There are different surgical methods for primary malignant tumor located at distal tibia. Previous studies have reported that double-strut fibula ankle arthrodesis is an alternative option. The purpose of this study was to investigate the biomechanical effect of double-strut fibula ankle arthrodesis by finite element analysis (FEA).

**Methods:**

Computer-aided design software was used to establish three-dimension models. Three different models were constructed: normal tibia-fibula-talus complex (model A), double-strut fibula ankle arthrodesis (model B), and reconstruction by ipsilateral fibula (model C). We used FEA to evaluate and compare the biomechanical characteristics of these constructs. Simulated load of 600 N was applied to the tibial plateau to simulate balanced single-foot standing. Output results representing the model von Mises stress and displacement of the components were analyzed.

**Results:**

Construct stiffness was increased when the internal plate fixation was used. For axial load, model B (1460.5 N/mm) was stiffer than the construct of model A (524.8 N/mm), and model C (636.6 N/mm), indicating model B was more stable. Maximum stress on the fibular graft occurred on the proximal end. The von Mises stress and stress distribution of fibular graft in model B (71.4 MPa) and model C (67.8 MPa) were similar. In model B, the ipsilateral fibula in model B has a higher value of stress (16.1 MPa) than that in model A (0.5 MPa), indicating the ipsilateral fibula shared load after fusion with talus.

**Conclusions:**

Our computational findings suggest that double-strut fibula ankle arthrodesis is an acceptable construct for distal tibia defect and the ipsilateral fibula shares load after fusion with talus.

**Supplementary Information:**

The online version contains supplementary material available at 10.1186/s13018-021-02362-0.

## Introduction

Primitive malignant neoplasm occurs at distal tibia is rare and typically has a better prognosis than other sites [1]. However, because the disease in this site is infrequent, the consensus of gold standard treatment has been not reached. Limb-sparing surgery consists of tumor resection and reconstruction using prosthesis, allograft, autograft, recycled tumor-bearing bone, or bone transport as well as ankle arthrodesis [2–9]. Each technique has pros and cons, and the best treatment modality is not clear. In a case series study, Zhao et al. [10] found similar limb function evaluated by Musculoskeletal Tumor Society (MSTS) scores between autograft reconstruction (81%) and amputation (82%), which were both superior to allograft reconstruction (67%). Meanwhile, the team has introduced a double-strut fibula ankle arthrodesis technique to restore limb continuity. A mid-term (53 ± 46 months) study conducted by the same team [11] in 2019 proved that double-strut fibula ankle arthrodesis is capable of achieving durable ankle fusion and limb function with low rate (11%) of complications.

Although promising clinical outcomes has been reported, whether double-strut fibula ankle arthrodesis is stable, and beneficial for patients still lack high-quality clinical follow-up and mechanical evidence. The purpose of this study was to investigate the biomechanical characteristics of double-strut fibula ankle arthrodesis by finite element analysis (FEA). Moreover, we compared this construct with normal bone model and the reconstruction using ipsilateral fibula and ankle arthrodesis. These biomechanical data may provide a theoretical reference for clinical treatment of bone defect of distal tibia.

## Materials and methods

**Fig. 1:**
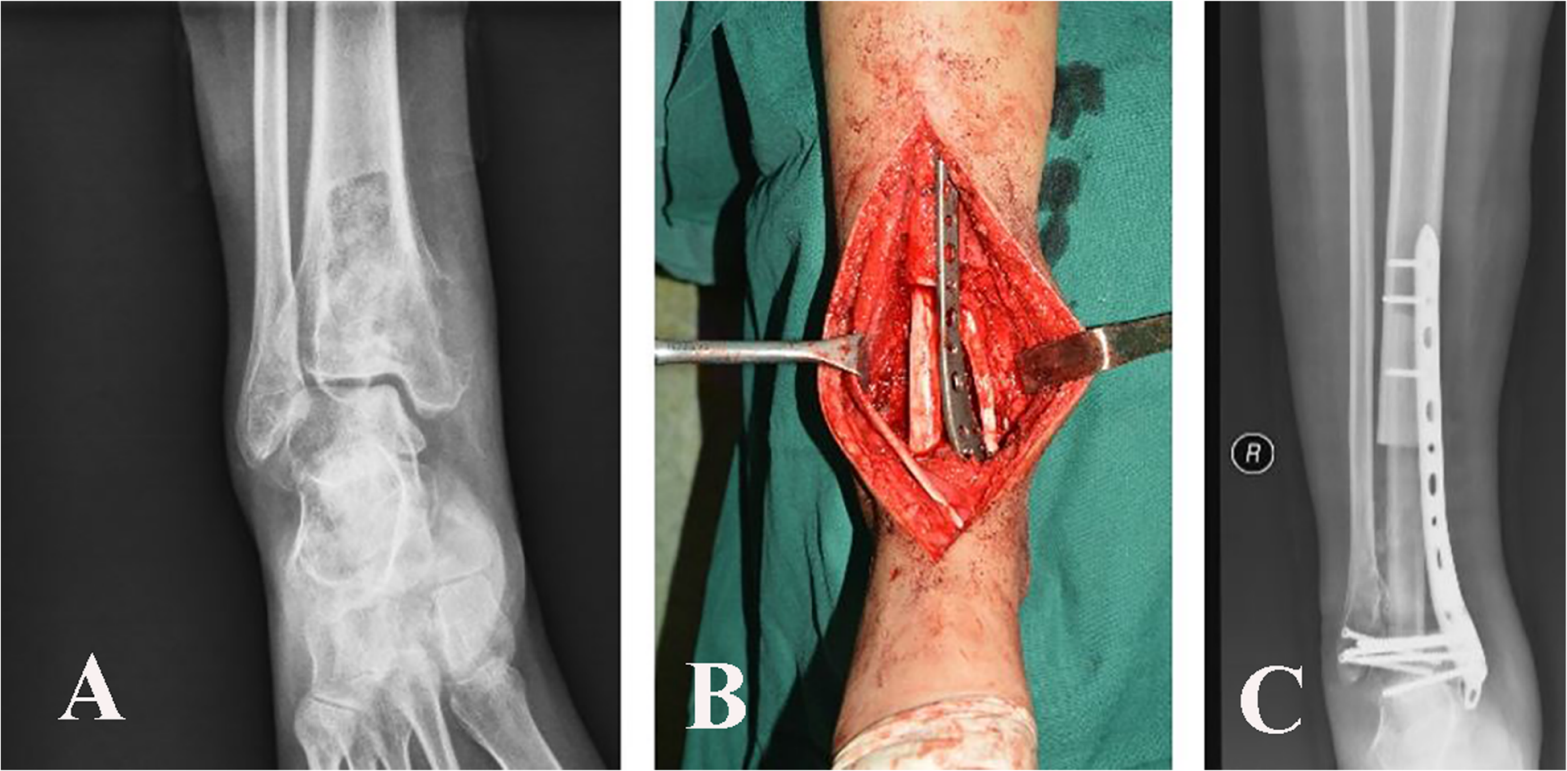
**a** Preoperative X-ray film shows osteosarcoma of distal tibia. **b** Intraoperative photo shows that a non-vascularized fibula transfer harvested from unaffected limb restore the tibial continuity, which parallels to the ipsilateral fibula. Fixation was carried out by plate and screws. The talus and ipsilateral fibula were fused. **c** Postoperative X-ray film

The tumor center of this study started to use the double-strut fibula ankle arthrodesis for malignant tumors of distal tibia in 2007 (Fig. 1). Surgical technique was introduced in previous study [11]. To 2020, a total of 9 patients with distal tibia tumors underwent tumor resection and this reconstruction. The resection length of distal tibia ranged from 7 to 20 cm, averaged 13 cm. Retrospectively, the mean follow-up duration was 53 months (standard deviation (SD), 46 months). The average bone union time of the proximal junction and distal junction was 10.5 months and 8.7 months, respectively. Reported postoperative limb function assessed by MSTS score system was 83%, ranged from 67 to 90%. There were no deep infection, plate breakage, or bone graft fracture during the follow-up time.

### Three-dimensional reconstruction models

**Fig. 2:**
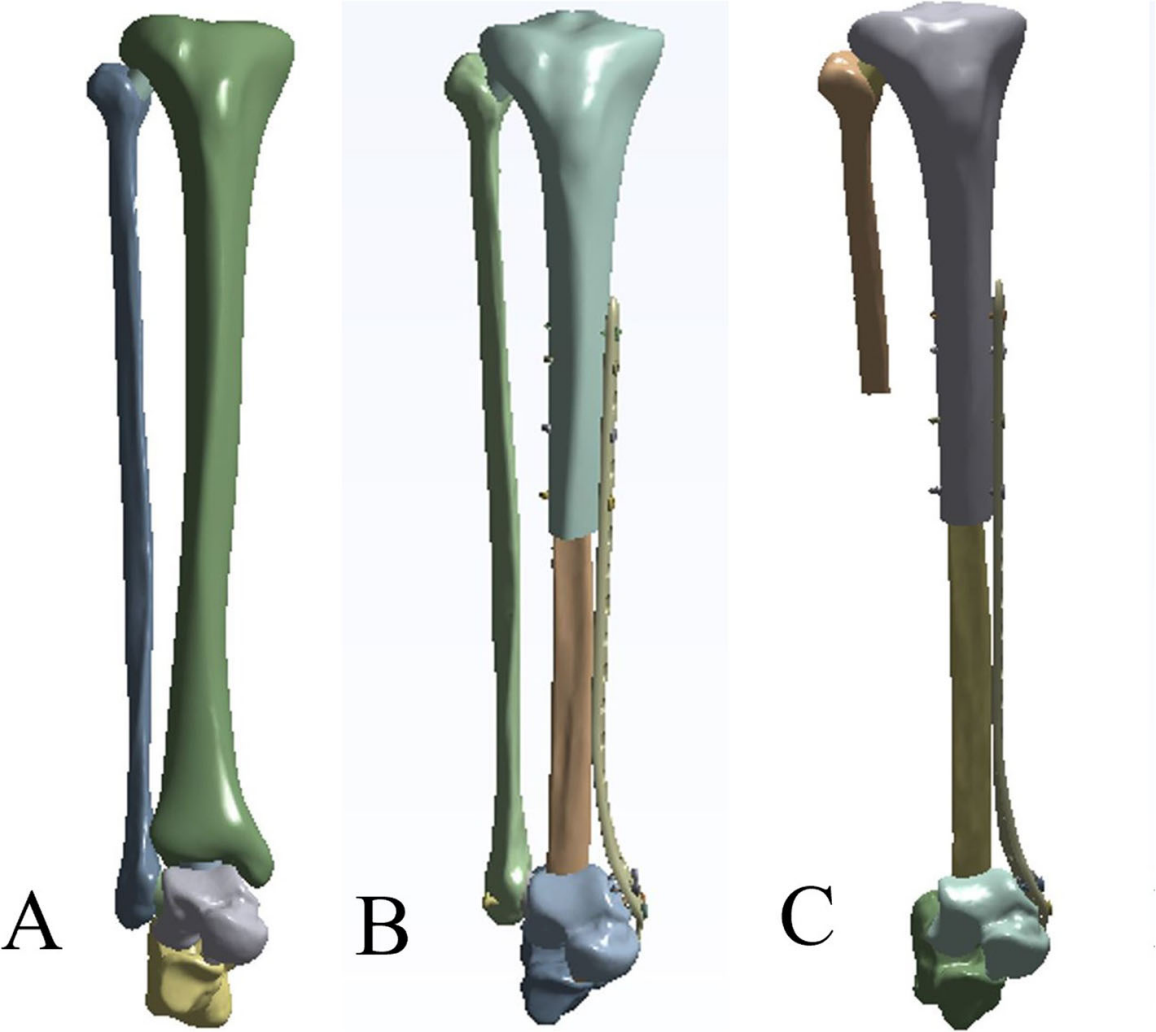
3D models for simulation calculation. (**a**) Normal tibia-fibula-talus complex, (**b**) double-strut fibula ankle arthrodesis, and (**c**) reconstruction by ipsilateral fibula—centralization of the ipsilateral fibula

A healthy adult male volunteer (age, 24 years; height, 168 cm; weight, 60 kg) was selected, and his right lower limb was placed in a neutral position to undergo computerized tomography (CT). Raw CT data was imported into the Mimics software (Materialise, Leuven, Belgium) to create a three-dimensional (3D) model. Definition of cortical bone, cancellous bone, and marrow cavity were established based on CT images with different gray values. Then 3D CT model data were obtained. This study simulated a distal tibial osteotomy of 13 cm according to previous clinical data. Then structure of each bone in IGS format was transferred to the Geomagics software (Raindrop Company, USA). Processing in the Geomagics software was done to obtain the volumes of the bones. Stp files of bone’s volume were imported into Solidworks software 2017 (Dassault Systemes Corp., French). Finally, the solid objects representing the bones were assembled using the software Solidworks (Dassault Systèmes Solidworks Corp., MA, USA) to make 3D tibia-fibula-talus complex. The appropriate computed model of plate and screw was supplied by Zimmer Inc. (USA). Finally, three different models (group 1) were assembled using Solidworks: normal tibia-fibula-talus complex (model A), double-strut fibula ankle arthrodesis (model B), and reconstruction by ipsilateral fibula (model C) (Fig. 2). In order to provide convinced data, in the same way, other two groups of models (groups 2 and 3) were constructed based on other two volunteers (170 cm, 60 kg; 165 cm, 60 kg). Namely, there are totally 3 model A, 3 model B, and 3 model C.

**Fig. 3:**
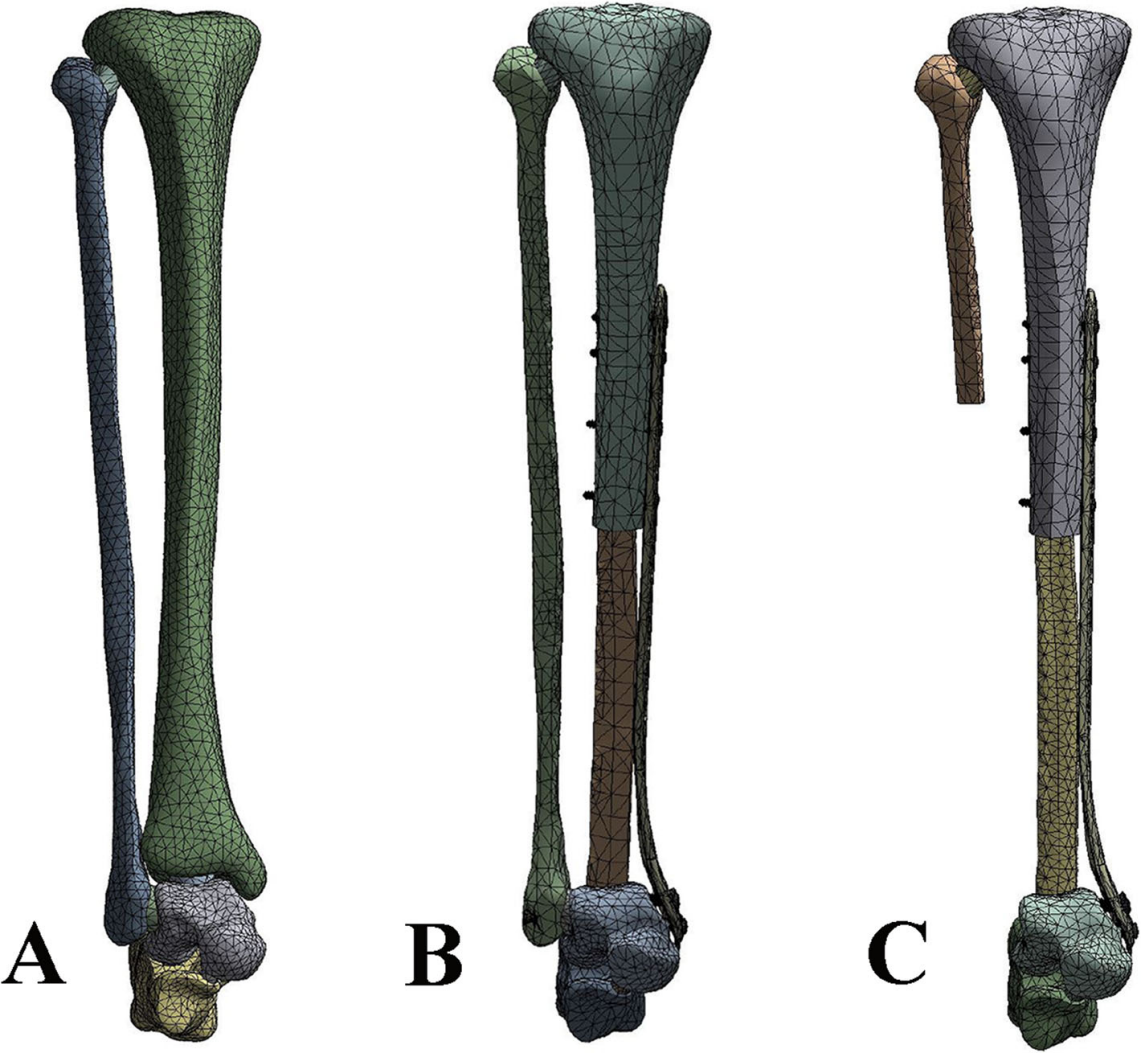
Tetrahedron volume-mesh models

3D models of reconstructions in SLDPRT files were then input into the FEA software Ansys 17.0 (Ansys Corp., USA). In Ansys, tetrahedron meshes of the models were created (Fig. 3). Interaction between the screw/bone interfaces and autograft/bone were defined as tie. The screws were fixed into the plates and tibia cortices and talus. All bones were assumed to behave as homogeneous, isotropic, and linearly elastic material. Material properties were assigned according to previous reports and were listed in Table 1. The cortical and cancellous portions of the distal tibia were modeled with Young’s modulus of 14,000 MPa and 700 MPa and Poisson ratio of 0.3 and 0.2, respectively [12–14]. Plate and screws were assigned an elastic modulus and Poisson’s ratio of 110,000 MPa and 0.3 respectively [15, 16].

**Table 1:** Material properties used in finite element models

Material	Young’s modulus (***E***)	Poisson ratio (***y***)
Plate, screw	110000	0.3
Cortical bone	14000	0.3
Cancellous bone	700	0.2
Fibula, talus, calcaneus	7300	0.3
Cartilage	3	0.4

### Boundary conditions and loading

The talus was fixed in all degrees of freedom. A vertical compressive force of 600 N corresponding the body’s weight of a person weighing 60 kg was applied to the tibial plateau in full extension to simulate balanced single-foot standing to bear the entire body’s weight. In the same way, other two groups of models were analyzed under the vertical loading of body’s weight (600 N), respectively.

Integral stability of the constructs was evaluated to compare construct stiffness. Regional stability of the constructs was assessed by exploring displacement at the fibula, fibular graft after fixation under axial loads. The von Mises stress (VMS) values and stress distribution on all components of the models were determined.

### Statistics

Statistical analyses were performed using the SPSS software (version 22.0; IBM Corp., Armonk, New York, USA). Descriptive statistics were used to determine means±standard deviations. The stiffness of the model was calculated by dividing the load by the vertical displacement of the model [17]. The stress distribution of the implant and fiblular graft was examined to speculate sites of stress concentration, respectively.

## Results

The mean number of nodes were 67702±19603, 763197±12446, and 654919±25613 in models A, B, and C, respectively, and mean amount of elements were 38521±12946, 45077±8352, and 387276±15213 in models A, B, and C respectively.

### Construct stiffness

**Fig. 4:**
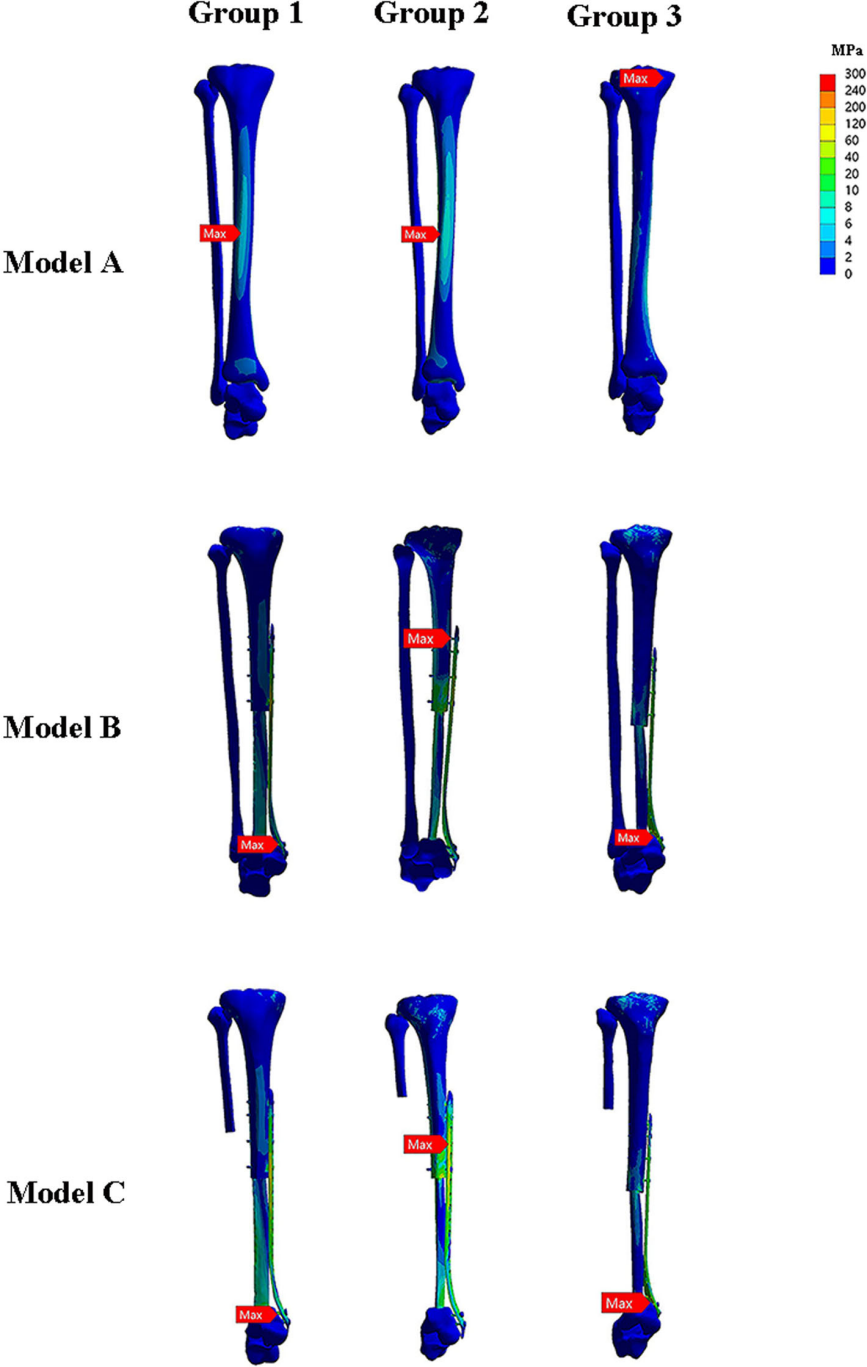
The pictures indicate stress distribution in the three groups of models when a vertical compressive force of 600 N corresponding the body weight of a person weighing 60 kg was applied to the tibial plateau

**Fig. 5:**
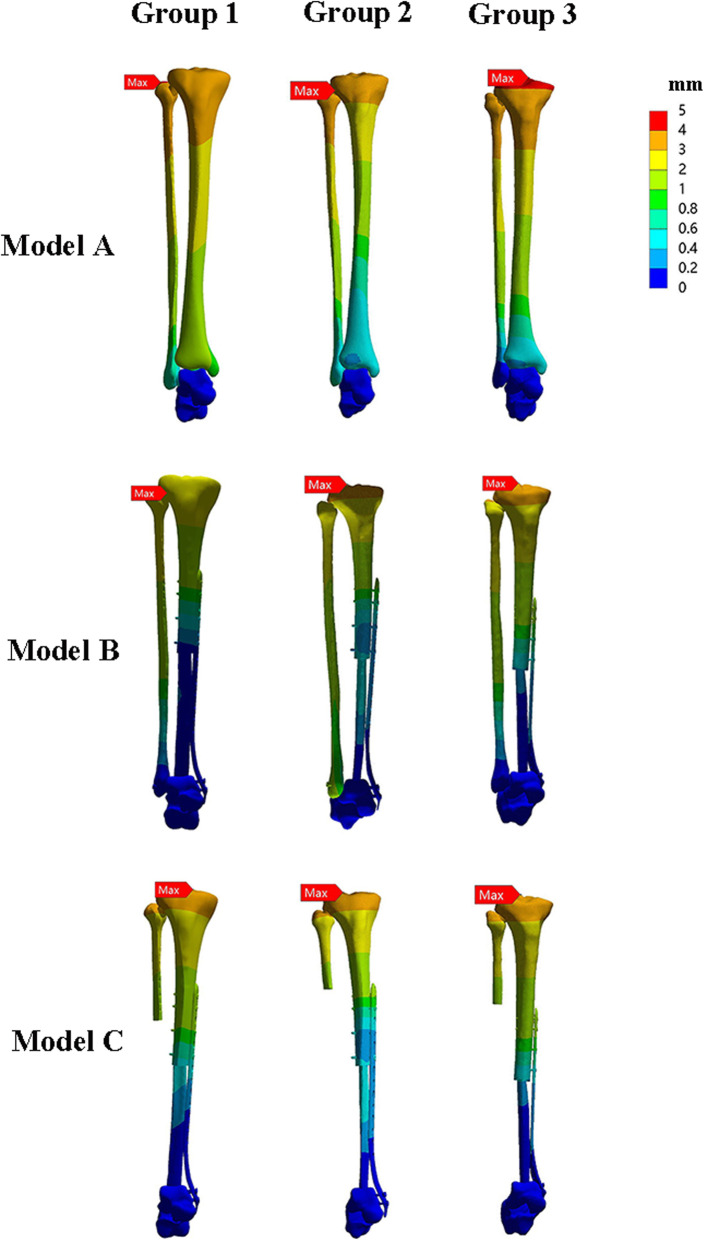
The pictures show the displacement distributions when a vertical compressive force of 600 N was applied to the tibial plateau

The VMS and stress distribution on three constructs were shown in Figs. 4 and 5. For axial load, model B (1460.5±1005.7 N/mm) was stiffer than the construct of model A (524.8±82.6 N/mm), and model C (636.9±135.5 N/mm). Overall, construct stiffness was increased when the internal plate fixation was used. However, the axial stiffness of double-strut fibular construct was approximately 2.3 times larger than that of the ipsilateral fibular reconstruction.

### Implant stress

**Fig. 6:**
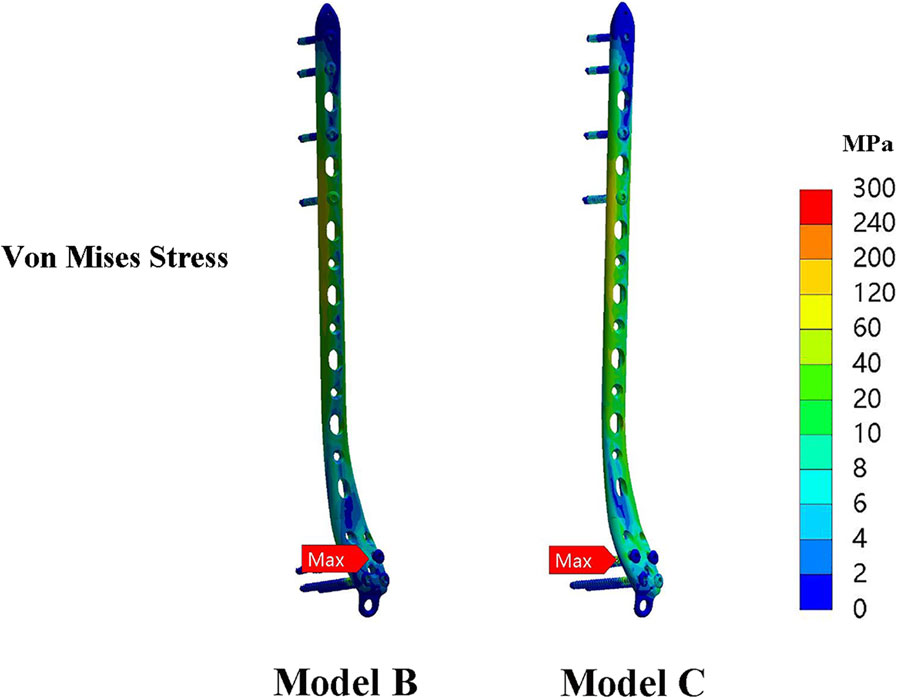
The maximum VMS of implant in model B was 203.14 MPa and was decreased by 13.8% compared to that in model C (235.6 MPa) (group 1)

For axial load, maximum stress on the implant occurred on the distal locking screw, and proximal locking screw, respectively. For model B, the ipsilateral fibular shared the load and decreased the risk of implant failure or graft fracture. The maximum VMS of implant after double-strut fibular construction (207.6±15.8 MPa) was decreased by 13.4% compared to that in model C (239.7±31.1 MPa) (Fig. 6).

### Graft stress and displacements

For axial direction, maximum stress on the fibular graft occurred on the proximal end. In regard to stress peak of the fibular graft, model B (71.4±45.5 MPa) and model C (67.8±39.8 MPa) provided similar stress distribution of fibular graft (Table 2).

**Table 2:** Maximum stress (MPa) of the three models when loading 600N

Component	Model A	Model B	Model C
Ipsilateral fibula	0.5±0.3 MPa	16.1±17.3 MPa	**/**
Fibular graft	/	71.4±45.5 MPa	67.8±39.8 MPa
Implant	/	207.6±15.8 MPa	239.7±31.1 MPa

**Fig. 7:**
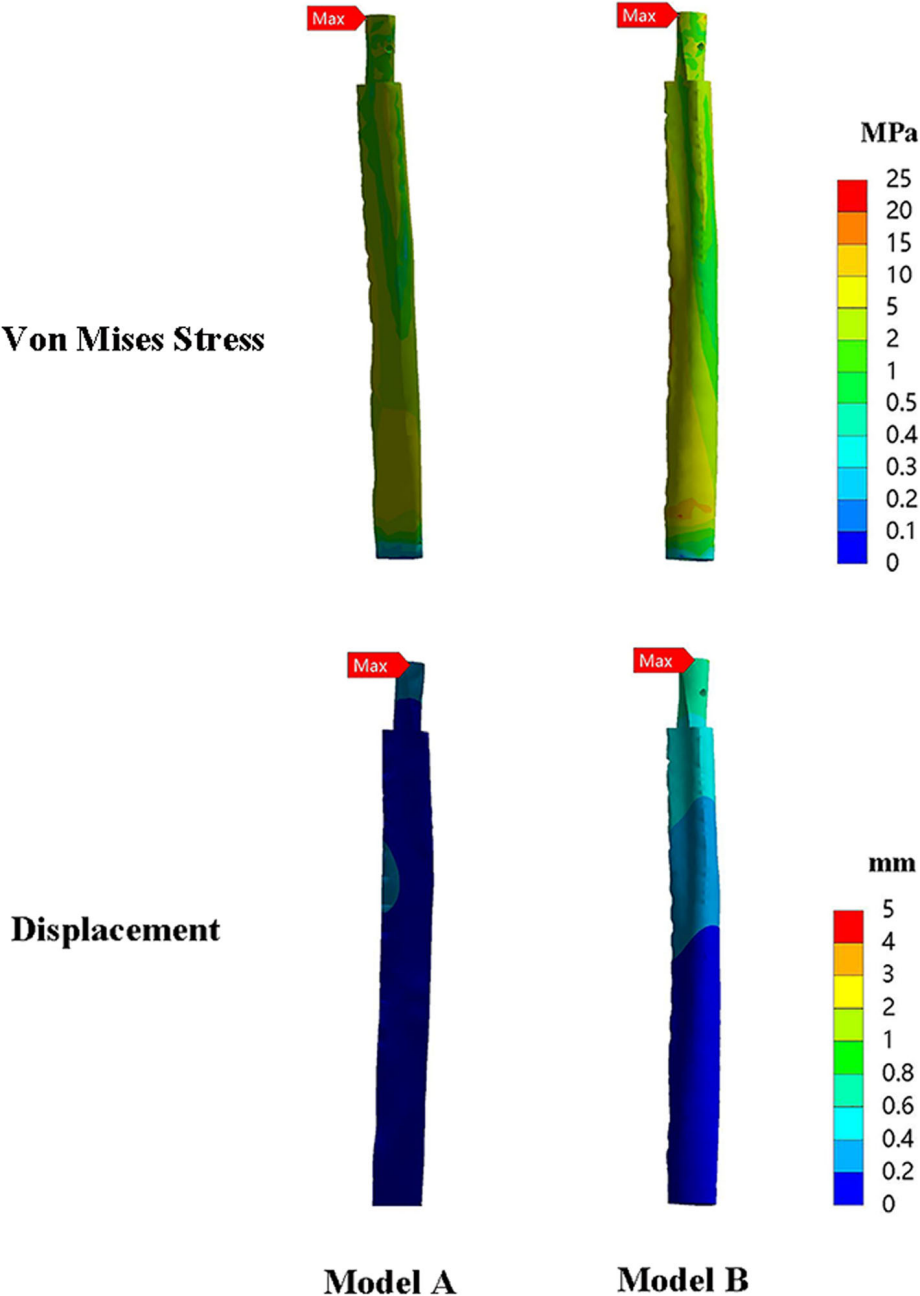
There were no obvious differences in VMS and stress distribution of fibular graft in model B (22.73 MPa) and model C (23.69 MPa). The maximum displacement at the fibular graft in model B (0.37mm) was less than that in model C (0.82 mm) (group 1)

Figure 5 shows the displacement values of three models. *Z*-axis represents the vertical displacement in direction of the applied load. The maximum of displacement of fibular graft was higher in model C (0.64±0.17 mm) than that in model B (0.48±0.10 mm)(Fig. 7).

### Stress and displacements of ipsilateral fibula

**Fig. 8:**
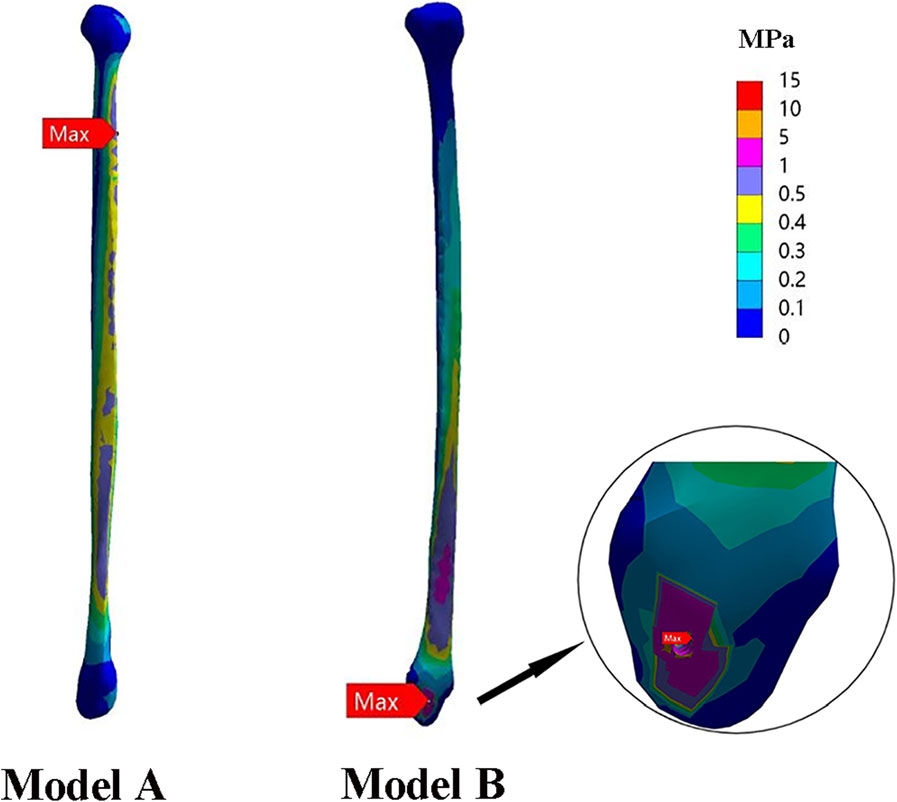
The ipsilateral fibula in model B has a higher value of stress (12.07 MPa) than that in model A (0.67 MPa), indicating the ipsilateral fibula shared load after fusion with talus (group 1)

In model B, the ipsilateral fibula was reserved and was fused with talus by a screw. Noteworthy, the result revealed that ipsilateral fibula in model B has a higher value of stress (16.1±17.3 MPa) than that in model A (0.5±0.3 MPa), indicating the ipsilateral fibula shared load after fusion with talus (Fig. 8).

## Discussion

In the literature, ankle fusion and reconstruction with bone graft is the primary method for salvaging the distal tibia [9]. The fibular graft is the widely used bone graft that is easy to obtain and results in minimal donor-site morbidity [18]. It can be inserted into the medullary canal of the tibia perfectly. Then, fibular graft has the ability to become hypertrophy under stimulation of weight-bearing after bony union [19, 20]. When this bone construction is created, initial stability is a guarantee of host-graft healing. However, it is difficult to investigate the biomechanical effect in vivo. Moreover, it is tough to enroll enough cases to detect the clinical outcomes of different surgical methods due to the rare incidence of this disease. As FEA has been widely used for mechanical analyses, it has the potential to predict the preoperative mechanical environment, help the surgeon to decide the optimal reconstruction. Therefore, we aim to evaluate the initial stability of double-strut fibula ankle arthrodesis.

This work has several limitations. First, the FEA model was based on the anatomy of three volunteers. Second, the role of muscles or ligaments was not simulated because of the difficulty in assessing the soft tissue changes after excision and reconstruction of the distal tibia. Therefore, the stability offered by the surrounding soft tissues was ignored. But, this technical limitation affected all the groups equally and it did not question the validity of our findings. And the mechanical results of this study are still credible because the three reconstruction methods were compared under the same simplified parameters. Third, it is a static simulated study and further studies are needed to explore the dynamic loading process. Finally, anatomical variations in the distal tibia and the extent of excision may affect the results.

According to the vertical pressure analysis, cracks usually occurred at the area of the concentrated stress and with obvious displacement. In this study, the maximum stress of fibular graft in model B and model C were similar (22.73 MPa vs. 23.69 MPa) when loading the vertical force of 600 N, which were both acceptable. In model B and model C, we noted that concentration of stress was at the implant. This can be easily explained by the fact that the plate can resist the upward displacement effectively and protect the fibular graft in early time. The outcome exhibited that fibula in model B has a higher value of stress (16.1 MPa) than that in normal bone model (0.5 MPa), indicating that the ipsilateral fibula acts as an ancillary structure for weight-bearing. It uphold the current recommendations that the double-strut reconstruction can provide satisfactory initial stability [10, 11].

The model C simulated the reconstruction method of ankle fusion with centralization of the fibula which was reported by Kundu et al. [7]. In previous study, 9 patients with distal tibia tumor underwent this surgical option, resulting in a mean MSTS score of 76%. There was no stress fracture of the fibula after surgery; however, an angulation at the proximal fibula graft was observed in one case. In the current study, we found that this technique of centralization of fibular graft was not stable enough compared with double-strut fibula reconstruction, and has a high rate of fracture of fibular graft in early period after surgery. Therefore, Kundu et al. [7] recommended that weight-bearing was not allowed in the first 8 weeks. Guarded weight-bearing was carried out 8-10 weeks onward when radiological bone union began, and the full-leg cast was replaced by a below-leg cast after 16-20 weeks, when radiographs showed sign of bone union. Therefore, this procedure requires quite a long time to get rid of cast and to start full weight-bearing.

Prosthesis can provide initial stability and good early function; however, it is associated with a significant set of complications such as high risk of infection, loosening, talus collapse, and ankle instability [9]. Due to lack of muscle coverage in this site, it will complicate the reconstruction of prosthetic replacement, and burden the prosthesis with long-term complications. A mid-term study and a long-term study exhibited that the aseptic loosening and infection were the main reason of prosthetic reconstruction failure [21, 22]. Zhao et al. [28] performed a literature review comparing prosthetic replacement with biological reconstruction (allograft or autograft), and revealed that autograft or allograft reconstruction performed better than prostheses. Therefore, in this study, we did not investigate the biomechanical effect of prosthetic replacement. However, in recent years, the introduction of 3D printed prosthesis with surface of bone growth may reduce the complications, further long-term study is needed.

The non-vascular autogenous fibular graft has some important advantages over other donor sites due to its length, geometry, and mechanical strength. The fibula being a long, straight tubular bone, with perfect shape allows tibial intramedullary insertion. And it is an easy, inexpensive biological procedure that does not require micro-vascular skills. The current FEA study suggest that reconstruction with fibular graft after tumor resection of distal tibia is an accepted solution, but the additional plating is required to sustain initial stability.

## Conclusion

The computational findings suggest that double-strut fibula ankle arthrodesis is an acceptable construct for distal tibia defect and the ipsilateral fibula shares load after fusion with talus.

## Supplementary Information

**Additional file 1: Figure 1.** The pictures indicate VMS of implant in three groups when a vertical compressive force of 600 N corresponding the body weight of a person weighing 60 kg was applied to the tibial plateau. **Figure 2.** The pictures indicate VMS of fibular graft in three groups.

## Data Availability

The datasets used in the study are available from the corresponding author on reasonable request.

## Abbreviations

FEA: Finite element analysis
MSTS: Musculoskeletal Tumor Society
SD: Standard deviation
CT: Computerized tomography
DICOM: Digital imaging and communications in medicine
3D: Three-dimension
VMS: Von Mises stress
