# Supplementary material for: Is double-strut fibula ankle arthrodesis a reliable reconstruction for bone defect after distal tibia tumor resection?—a finite element study based on promising clinical outcomes
Source: J Orthop Surg Res. 2021 Mar 29;16:230. doi: 10.1186/s13018-021-02362-0 (PMC8006370; doi:10.1186/s13018-021-02362-0)
Supplement: Supplementary file 1 — Additional file 1: Figure 1. The pictures indicate VMS of implant in three groups when a vertical compressive force of 600 N corresponding the body weight of a person weighing 60 kg was applied to the tibial plateau. Figure 2. The pictures indicate VMS of fibular graft in three groups. [file 13018_2021_2362_MOESM1_ESM.docx]

**1. The Models**

**1.1 Group 1 (height, 168 cm; weight, 60 kg)**


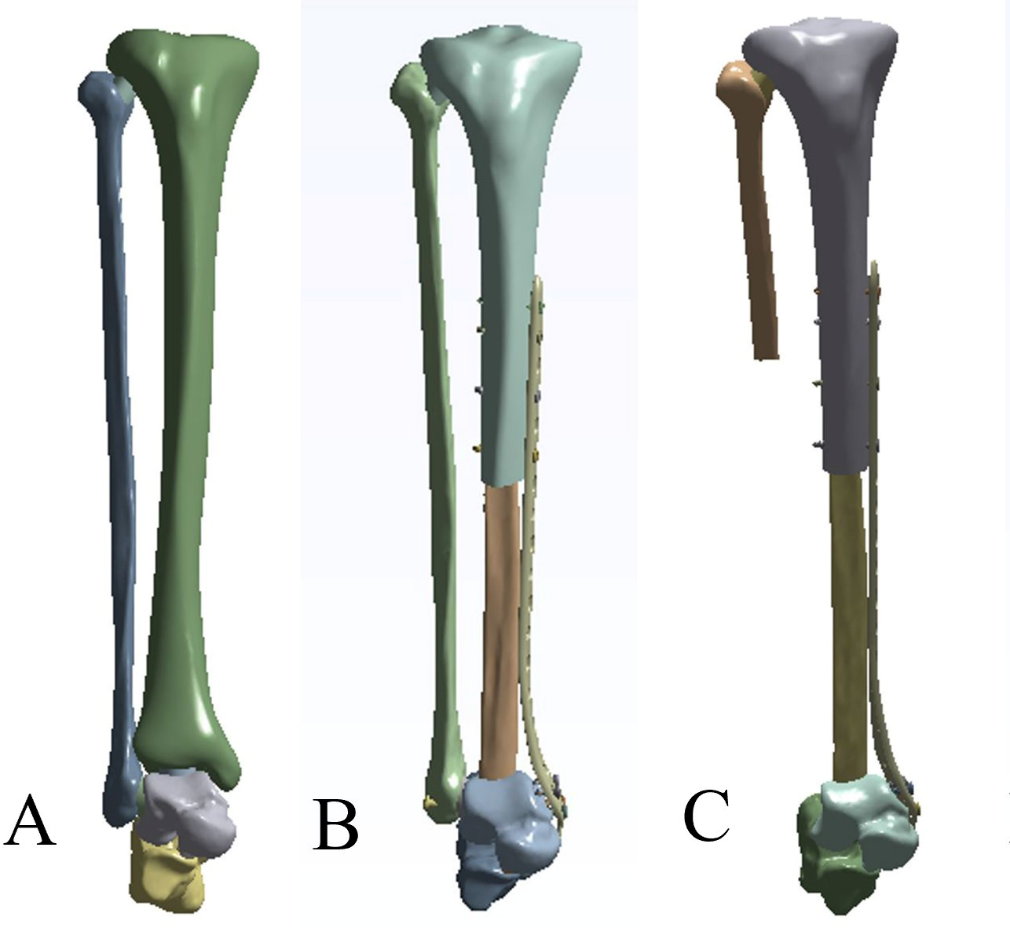


**1.2 Group 2 (height 170cm; weight 60kg)**


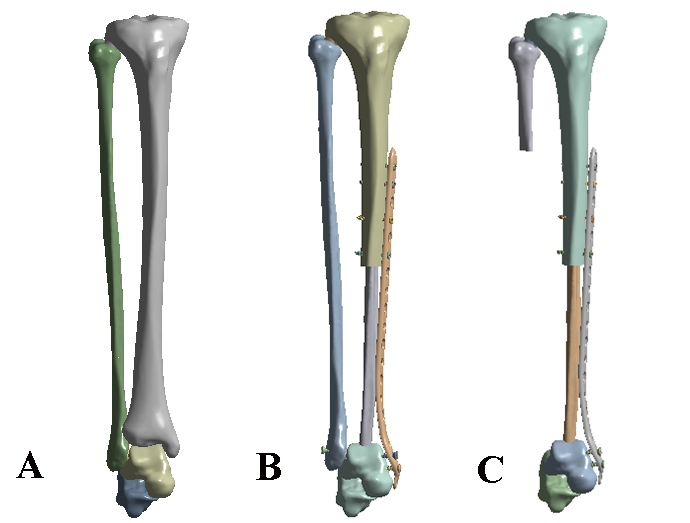


**1.3 Group 3 (height 165cm; weight 60kg)**


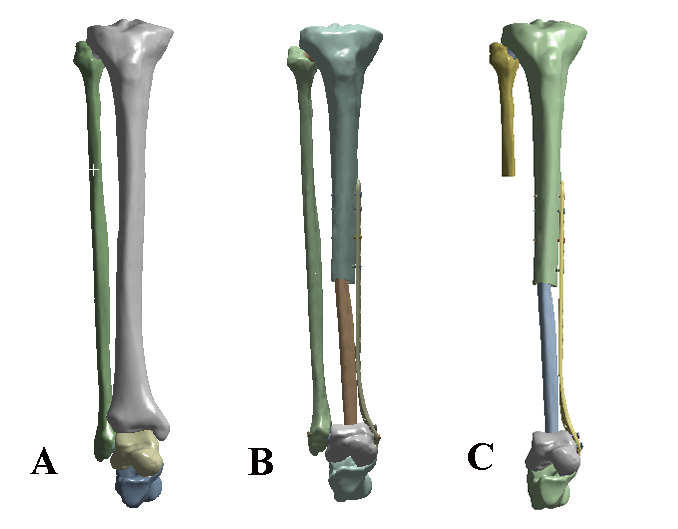


**2. Supplemental Figures**

**
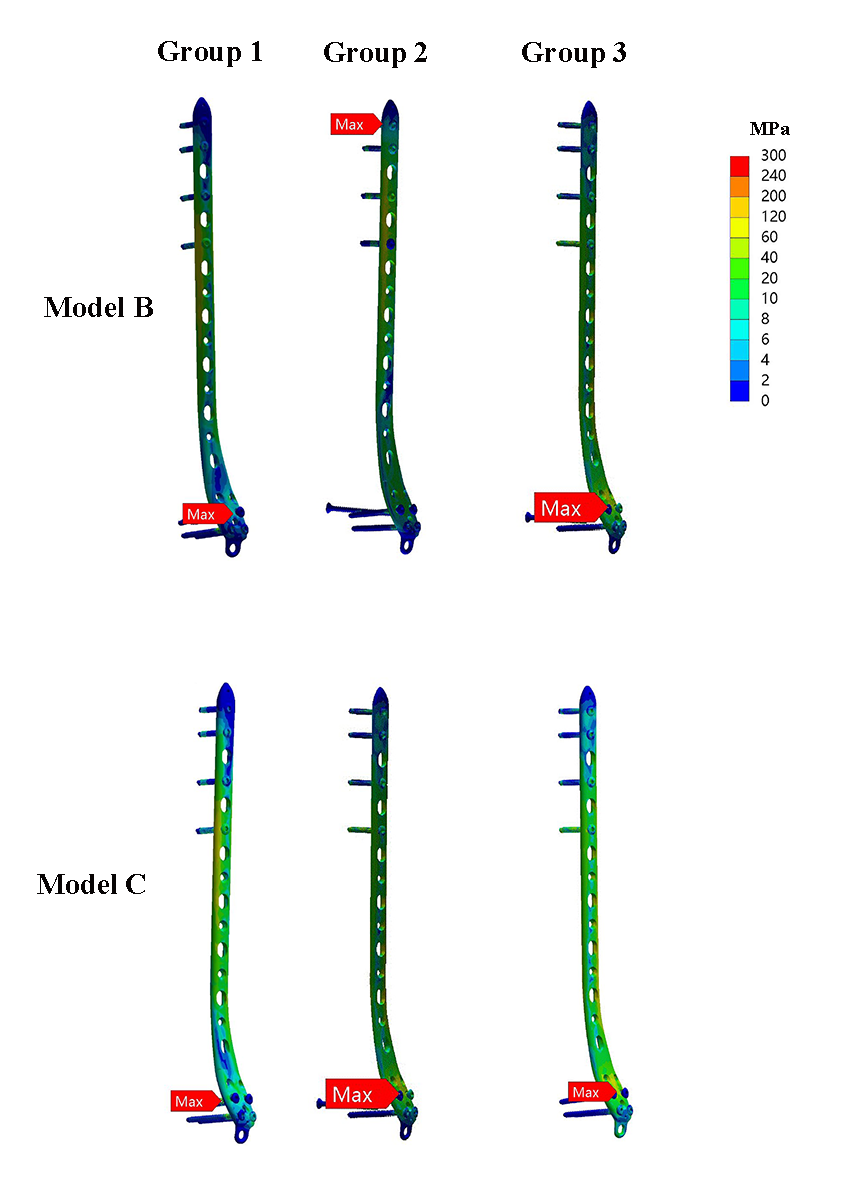
**

**Figure 1** The pictures indicate VMS of implant in three groups when a vertical compressive force of 600 N corresponding the body weight of a person weighing 60 kg was applied to the tibial plateau.

**
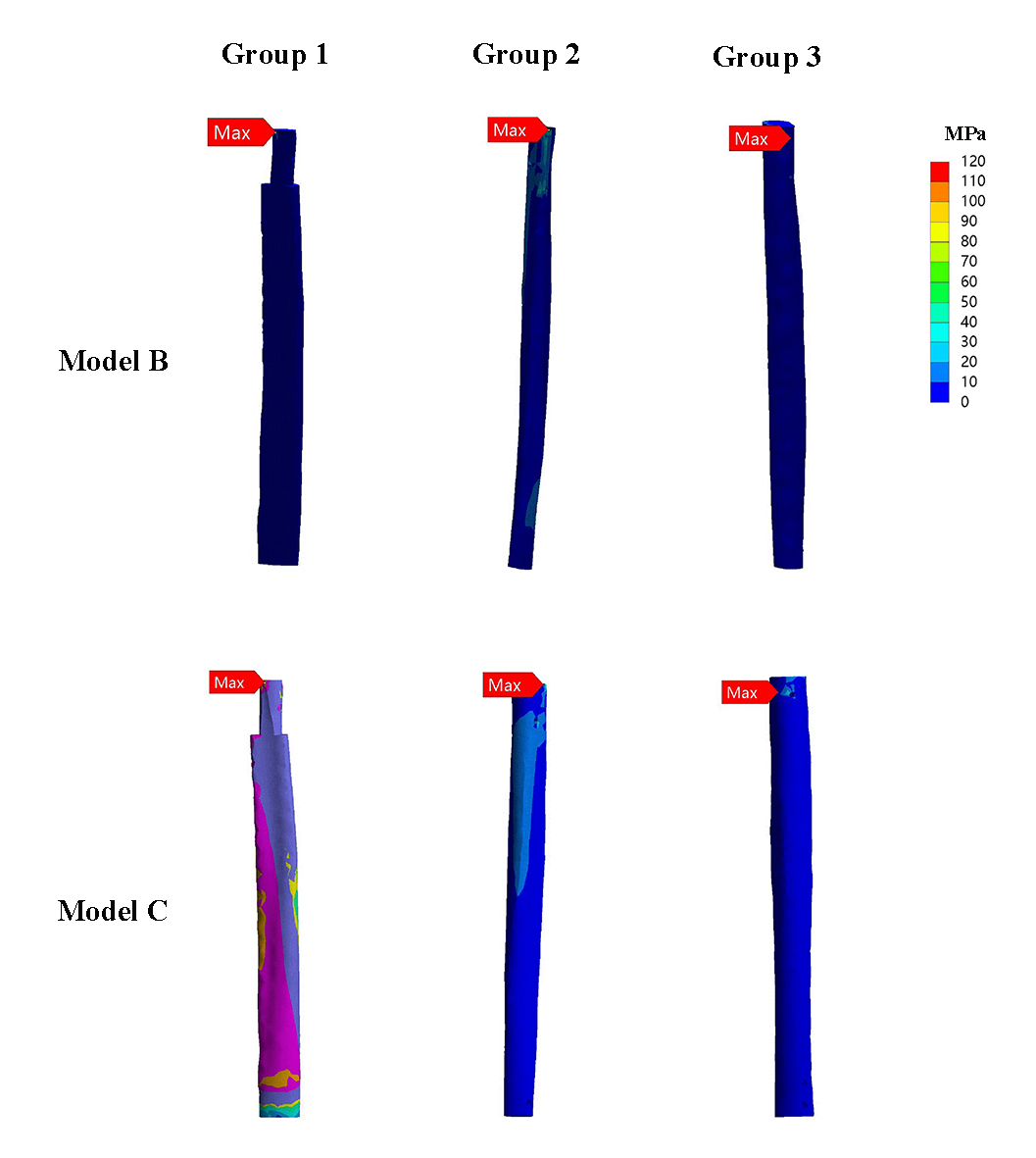
**

**Figure 2** The pictures indicate VMS of fibular graft in three groups.
